# Sex disparities in the prevalence of physical function disabilities: a population-based study in a low-income community

**DOI:** 10.1186/s12877-021-02362-z

**Published:** 2021-07-10

**Authors:** Elsa M. Orellano-Colón, Erick L. Suárez-Pérez, Marta Rivero-Méndez, Claudia X. Boneu-Meléndez, Nelson Varas-Díaz, Mauricio Lizama-Troncoso, Ivonne Z. Jiménez-Velázquez, Arelí León-Astor, Jeffrey W. Jutai

**Affiliations:** 1 Occupational Therapy Program, School of Health Professions, University of Puerto Rico, Medical Sciences Campus, PO Box 365067, San Juan, PR 00936-5067 USA; 2 Department of Biostatistics and Epidemiology, Graduate School of Public Health, University of Puerto Rico Medical Sciences Campus, San Juan, USA; 3 School of Nursing, University of Puerto Rico Medical Sciences Campus, San Juan, USA; 4 Global and Sociocultural Studies, Florida International University, Florida, USA; 5 Puerto Rico Assistive Technology Program, University of Puerto Rico Central Administration, San Juan, USA; 6 School of Medicine, University of Puerto Rico Medical Sciences Campus, San Juan, USA; 7 Office of Environmental Quality, Health, and Occupational Safety, University of Puerto Rico Medical Sciences Campus, San Juan, USA; 8 Interdisciplinary School of Science, University of Ottawa, Ottawa, Ontario Canada

**Keywords:** Activities of daily living, disability, Frail elderly, Hispanic, Multiple chronic conditions, Sex

## Abstract

**Background:**

Functional disability continues to be a significant public health problem that increases older adults’ vulnerability to experience a diminished quality of life, loss of independence, higher healthcare costs and health services utilization, and increased risks of mortality. Thus, we aimed to study the prevalence of functional disabilities by sex according to the types of daily living activities, controlling for specific sociodemographic variables among older Hispanics from low-income communities.

**Methods:**

We used a cross-sectional epidemiological research design, considering a complex sampling design of households to interview adults ≥65 years living in low-income communities in Puerto Rico. Functional disability was measured by the PROMIS® Physical Function Short Form-20 T-score. The selected community was reported to have 5980 adult residents ≥65 years, according to the USA Census. The prevalence of functional disability was estimated using the logistic regression model, weighting by the effect of the sampling. Our estimated prevalence was compared between sexes using the prevalence ratio (PR), which was estimated with logistic regression models, controlling for age, income, number of chronic conditions, high and low impact of chronic conditions in functional disabilities, marital status, and sampling design.

**Results:**

We recruited 211 older Hispanics from a randomly selected sample. Their mean age was 74.4 ± 7.1 years, with female predominance (57.3%). The overall estimated prevalence of physical function disability using T-score among females was 2.70 (95% CI: 1.4, 5.1) times the estimated prevalence of physical function disability among males. Women were more likely to report functional disabilities in instrumental activities of daily living, self-care activities, and functional mobility compared to males. However, sex differences were largely explained by the presence of musculoskeletal conditions of high impact in functional disability.

**Conclusions:**

The females in our study bear the greater burden of physical function disability in their adult age. Health policies, as well as future studies, should be targeted at reducing the burden of physical function disabilities in different types of daily activities through gender-sensitive disability self-management programs.

## Background

Functional disability defined as any difficulty performing activities of everyday life that are essential to independent living [1], is a major adverse outcome of age-related chronic conditions such as arthritis, chronic back pain, cardiovascular diseases, and diabetes [2]. Functional disabilities continue to be a significant public health problem that increase older adults’ vulnerability to experience diminished quality of life, loss of independence, higher healthcare costs and health services utilization, and increased risks of mortality [3–5].

Functional disabilities do not occur uniformly across races and ethnicities. According to the U.S. Census Bureau [6], older Hispanics (65 years and older) living in Puerto Rico reported a striking higher number of functional disabilities in daily activities (29.9%) as compared to older Hispanics (20.6%) and older Whites (15.0%) living elsewhere in the United State of America (USA). Puerto Rico was followed by West Virginia with a prevalence of 17.6%. Moreover, functional disabilities disproportionally affect males and females. Although older females experience higher life expectancy, they consistently report more functional limitations and physical disability than their male counterparts [7–10]. This is particularly relevant for the older population in PR, in which older females suffer from more frequent functional disabilities in independent living (30.7%) as compared to males (21.4%) [6]. Functional disabilities may be more common among older females for several reasons. Some researchers attribute this higher prevalence of disability among females to their lower rates of mortality and recovery from disability onset, resulting in longer durations of disability [11]. More severe functional disabilities among females have also been explained by having more physical chronic conditions that affect functioning. Specifically, females in Puerto Rico have been shown to have a higher prevalence of debilitating conditions such as cartilage and bone disorders (35.0% vs 10.7% for older males) [12], joint pain (24.0% vs. 16.5%) [12], asthma (89 .5% vs 10.5%) [12, 13], and arthritis (26.2% vs 15.5%) [12, 13]. It also has been published that difference may arise because of innate anatomical differences between males and females which tend to give males an advantage in physical performance such as greater strength, mobility, and speed [14]. Researchers have also questioned whether the differential functioning of males and females reflects reporting differences. Evidence from several studies suggest that females are socialized to acknowledge their illnesses where males are socialized to be the “tougher” sex, that may result in fail to admit to physical weakness [15–20].

While there is a substantial amount of literature that describes sex differences in functional disabilities [7–10], studies that have specifically examined sex differences in self-care, instrumental activities of daily living (IADL), and functional mobility disabilities are more limited, particularly those that have attempted an in depth analysis aimed at controlling for sociodemographic factors such as age, health conditions, and income with the prevalence of functional disabilities across sexes in Caribbean populations. Given that females with functional disabilities made up a substantial larger share (30.7%) of the populations of those 65 years and older reporting functional disabilities in independent living activities as compared to males (21.4%) [6], studying the prevalence of functional disabilities by sex according to the types of daily living activities, controlling for specific sociodemographic variables, is an important question for the design of preventive and management programs for the population of community-living older adults. Therefore, the aims of this study were to: (1) determine the prevalence of functional disabilities among Hispanics 65 years and older and (2) compare the prevalence of functional disabilities in specific self-care activities, IADL and functional mobility between males and females when controlling for age, income, chronic conditions, marital status, and sampling design.

## Methods

To achieve these aims we implemented a cross-sectional epidemiological design, considering a complex sampling design of households to interview older adults living in eight low-income communities in Puerto Rico.

### Study design and sample

The data collection was performed from November 2019 to March 2020 from the area known as *Caño Martín Peña* (CMP) located in the north of San Juan, PR. The CMP comprises eight poor and disadvantaged communities grouped by the PR Law 489–2004, to guarantee citizen participation processes that result in an enhanced quality of life of over 20,000 residents of these communities. Almost one third of these communities are older people living with chronic health conditions.

Initially, we identified the numbers of households blocks within the census tracks (CTs) of the CMP. Then, we identified the number of occupied households within the blocks according to the information provided by the most recent Census data. Afterwards, we conducted a random selection of thirty-five (35) blocks of households from all census tract within the CMP. Four blocks at census tract 46 (blocks 5006, 5009, 5000, and 5010) were excluded from this selection because they fall outside the boundaries of the CMP district. In each selected block, the households were grouped in 4 consecutive occupied households to determine a segment of households. One segment was randomly selected in each block to reach the number of participants needed per block. Due to the rejection rate, we intended to reach at least ten eligible subjects per block. Table 1 shows the expected number of subjects to be recruited in each census tract:

**Table 1:** Number of subjects to be recruited in each census tract

Census Tract	Expected number of subjects to be recruited in each CT	Number of occupied households	Expected number of segments^1^
**36**	26	13	3
**37**	71	35	9
**38**	32	16	4
**44**	34	17	4
**45**	43	21	5
**46**	84	41	10
Total	290	143	35

^1^ 1 segment = 4 occupied households

Once the segments were identified in geographical maps, the research assistant visited the area to visually confirm the occupied households. In all these visits, we were accompanied with the community leaders.

### Recruitment procedures

Five community interviewers were selected to conduct the recruitment and data collection. These interviewers received in November of 2019 a 4 days face-to-face training by an occupational therapist, a nurse, a psychologist, and an epidemiologist. The recruitment process was performed in several visits. During the first visit, the community interviewers dropped off a letter and a flyer about the study. On the second visit, they knocked on the door of the selected homes. If nobody answered, a second letter was dropped off, followed by an additional visit. The time and day of the week for these additional visits systematically varied to maximize the chance of contact. When contact was made, the community interviewers identified a person in each household who met the inclusion criteria of the study: (1) Spanish speaking Hispanic adults ≥65 years, (2) living independently at home in the CMP community, and (3) able to provide informed consent and participate in an interview evidenced by a score ≥ 12 in the Caban Minimental State Examination test [21]. Participants were excluded if they received home health care services, required supervision to perform their activities of daily living or were bedridden. These criteria were designed to enroll older adults with the potential of having some functional disability but who were not totally dependent or homebound or receiving services to address functional problems. All eligible subjects at the visited household were invited to participate in the study. The community interviewers filled a contact information form and scheduled a meeting in the homes of those who met the first two inclusion criteria and showed interest in participating. During this meeting, the community interviewers explained the purpose, content and procedure of the study, duly following the informed consent process. Those who agreed to participate, signed the informed consent and completed the Cabán Minimental State Examination test, the last inclusion criteria. Those who passed this test completed the socio-demographic questionnaire, followed by the PROMIS® Physical Function Short Form-20 [22]. Study participation was entirely anonymous (no personal identifiers were collected) and all participants received a $25 incentive for their participation.

### Study variables

#### Outcomes

Functional disability which is the respondent’s perceived ability to perform a variety of physical activities patient-reported outcome measure. We used the PROMIS® Physical Function Short Form-20*,* developed as part of the PROMIS initiative, the largest effort worldwide to improve patient-reported (PRO) measures [22]. The PROMIS® Physical Function Short Form-20 is designed to estimate the respondent’s perceived ability to perform a variety of physical activities on a 5-point Likert-type scale ranging from 5 “without difficulty” to 1 “unable to do”; adding the scores of each physical activity, we defined a raw score [22]. Afterward, we converted the raw score to a T-score, which is a standardized score with a mean of 50 and a standard deviation (SD) of 10. Higher T-scores indicate greater physical function. This measure has demonstrated sound psychometric properties when implemented in people with physical impairments, such as back conditions, neck conditions, and multiple sclerosis [23–25]. The PROMIS physical functioning items were translated into Spanish using a universal approach for translations and cultural adaptation of instruments [26–28] and has demonstrated an excellent high internal consistency (Cronbach’s alpha = 0.91) with the English physical function items [29].

We also defined if the participant had disability in each type of physical function disability (IADL, self-care and functional mobility) according to the items from the raw score of PROMIS Physical Function Form. If the participant’s score was below the median in each category, they were identified as having a physical function disability. For example, if the participant score ≤ 23 in IADL, they were identified with a physical function disability.

#### Main predictor: sex

Sex was categorized using self-reported questionnaire.

#### Confounding variables

We used a sociodemographic questionnaire developed for the purpose of this study to collect participants’ self-reported sociodemographic data. The potential confounding variables included in this study were: (i) age (≤ 74 years and > 74 yeas), (ii) sex (male and female), (iii) marital status (married/having a partner, single/separated/divorced, widowed), (iv) educational level (less than high school, high school graduate or GED diploma, some college or greater), (v) working status (retired, disabled, housekeeper, employed), (vi) annual income (less than $5000, $5000 - $9999, $10,000 - $14,999, $15,000 - $24,999, $25,000 - $34,999, $35,000 - $49,999, $50,000 - $74,999), (vii) sources of income (social security, pension, public assistance [nutritional assistance, public welfare program], (viii) Veteran’s benefit, earnings [full-time work, part-time work, or informal earning), and (ix) healthcare coverage (Medicare, government healthcare, other), (x) poverty level (annual income below and above $10,000). These confounding variables were selected based on evidence that age, sex, income, marital status, and economic status may have an effect on the presence of functional disabilities [30–32].

#### Effect modifier

The type of chronic conditions, identified using self-report of medical conditions status during the data collection phase, was considered as a potential modifier effect in the relationship between sex and functional disability. Since there is some evidence supporting that musculoskeletal conditions such as arthritis, osteoporosis, and chronic back pain are more disabling for females than for males [9, 33–36], we regrouped the chronic conditions into two groups: high impact and low-moderate impact on functional disability. This is, high impact on functional disability included arthritis, low back pain, and osteoporosis. Low-moderate on functional disabilities included high blood pressure, obesity, heart disease, gastrointestinal disorder, respiratory disease, visual, hearing, depression, anxiety and other.

### Sample size determination

The original sample size was 250 residents of CMP who were 65 years old or older.

This number was computed based on a prevalence of 5980 adults residents ≥65 years living in the selected community in 2010, according to the USA Census, (as reported in Sheffield et al. [37]) and the precision approach for the prevalence estimation of functional disability. This computation was made under the assumption that this prevalence was 30%, (based on previous studies in Puerto Rico [38]) and a confidence level of 95% to reach a margin of error of 5.7% (1.96*√(.3*.7/250)). However, due to the government lockdown during the COVID-19 pandemic, the final sample size was composed of 211 participants. As a consequence, with *n* = 211 and 95% confidence level, the estimated margin of error may increase to 6.2% using the previous reported prevalence (30%) or to 6.7% using the current prevalence of disability (58%). On the other hand, the statistical post-power to assess sex differences of functional disability, using the current prevalence estimation per sex with *n* = 211 and 5% significance level, was greater than 90%.

### Statistical analysis

Initially, we performed an epidemiological profile of the study participants at CMP. We used measures of central tendency such as median, mean and percentile. The Fisher’s exact test was used to assess these associations between sex and different clinical and demographics characteristics, considering different disability indexes. The prevalence of functional disability and chronic conditions was estimated with 95% confidence intervals using the logistic regression model, weighting by the effect of the sampling design (relationship of subjects in the study population per each sampled subject). Afterward, the excess in the prevalence by sex (Females vs Males) was estimated using the prevalence ratio (PR).

The PR was estimated using a logistic regression model with 95% confidence intervals, controlling the effect of different potential confounders and the sampling design. The effect of the sampling design was defined with the following weighting factor:

$$ weigh{t}_{ij}=\left(\frac{1}{p{s}_{ij}}\right)\ast \left(\frac{1}{p{ar}_j}\right) $$

where *weight*_*ij*_ indicates how many persons in the target population were represented by each selected person in the *i-th* block within the *j-th* census tract; *ps*_*ij*_ indicates the probability of selection the *i-th* block in within the *j-th* census tract and *par*_*j*_ indicates the participation rate in the *j-th* census tract. Before the PR was adjusted by different potential confounders, we assessed the statistical significance of different interaction terms in the logistic model using the likelihood ratio test. All statistical programming was performed using STATA (Version 14.1, College Station, TX, USA). The significance tests were assessed with *p*-value < .05.

## Results

### Participation and eligibility

Up to March 16, 2020 we were able to approach 335 potential participants. Among these, 103 did not met one or more of the inclusion criteria, 18 refused to be screened, and 214 were recruited. From these, three refused to participate resulting in an analytical sample size of 211 participants; 121 females and 90 males. Table 2 shows the sociodemographic characteristics of the participants.

**Table 2:** Demographics characteristics by sex (*n* = 211)

Charac-teristics	Category	Total	Sex		*p*-value^#^
			Female (n_f_ = 121)	Male (n_m_ = 90)	
Age (years)	≤74	115 (100%)	66 (57%)	49 (43%)	> 0.1
	75+	96 (100%)	55 (57%)	41 (43%)	
Academic achievement	Less than High School	137 (100%)	72 (53%)	65 (47%)	> 0.05
	High School Graduated	44 (100%)	28 (64%)	16 (36%)	
	Some College or Greater	30 (100%)	21 (70%)	9 (30%)	
Marital status	Married or Partner	90 (100%)	45 (50%)	45 (50%)	0.069
	Single, separated, divorced or widowed	121 (100%)	76 (63%)	45 (37%)	
Number of persons at home	Live alone	72 (100%)	41 (57%)	31 (43%)	> 0.01
	2 persons	103 (100%)	62 (60%)	41 (40%)	
	More than 2	29 (100%)	15 (52%)	14 (48%)	
Annual income	Under $5000	47 (100%)	29 (62%)	18 (38%)	> 0.01
	$5000–$9999	90 (100%)	57 (63%)	33 (37%)	
	$10,000–$14,999	40 (100%)	19 (48%)	21 (53%)	
	Over $15,000	29 (100%)	13 (45%)	16 (55%)	
Work status	Retired	112 (100%)	50 (45%)	62 (55%)	< 0.0001
	Disabled	26 (100%)	14 (54%)	12 (46%)	
	Full time home (Homemaker)	55 (100%)	54 (98%)	1 (2%)	
	Employed	15 (100%)	2 (13%)	13 (87%)	
Source of income	Retirement	35 (100%)	18 (51%)	17 (49%)	0.049
	Social Security	180 (100%)	104 (58%)	76 (42%)	
	Nutritional Assistance Program	83 (100%)	60 (72%)	23 (28%)	
	Others^a^	6 (100%)	5 (83.3%)	1 (18.7%)	
Self-identification of the ethnic group	Puerto Rican	180 (100%)	106 (59%)	74 (41%)	0.090
	Dominican	28 (100%)	12 (43%)	16 (57%)	
	Other	3 (100%)	3 (100%)	0	
Health care plan	Medicare	153 (100%)	89 (58%)	64 (42%)	> 0.01
	Government	37 (100%)	23 (62%)	14 (38%)	
	Other	21 (100%)	9 (43%)	12 (57%)	

^a^ Others includes Veteran, TANF Working (full-time, part-time, informal)

# Using Fisher exact test to compare sex distribution

Approximately, half (53.7%) of participants were female, the overall mean age was 74.4 years (± 6.8). Males were reported to have less educational attainment than females; among the participants with high school or with some college degree, 70% were females. Among the participants who were married or living with a partner, females and males were equally distributed; however, among those who were single, separated or divorced, 63% were females. Among the participants with an annual income under $5000, 62% were females; however, the participants with an annual income over $15,000, 55% were males. The working status showed significant difference by sex (*p* < 0.0001); among the participants who has employment, 87% were males. Also, the source of income by sex showed significant difference (*p* = 0.049). Among the participants receiving Nutritional Assistance, 72% were females. The participants receiving Social Security, approximately 58% were females. Among the participants receiving Medicare, 58% were females. Among the participants receiving Government health care plan, 62% were females.

Our results showed different prevalence of chronic conditions between sexes (See Table 3). The prevalence 4 or more comorbidities was statistical different by sex (*p* = 0.0003); the estimated prevalence among females was 76.9%, and among males was 53.3%. Females showed higher prevalence, mainly in the following conditions: osteoporosis, gastrointestinal disorder, arthritis and respiratory disease. In contrast, males showed higher prevalence, mainly in the following conditions: hearing conditions (*p* = 0.04), and anxiety (*p* = 0.013).

**Table 3:** Prevalence^a^ of clinical conditions by sex (*n* = 211)

Conditions	Overall *n* = 211	Sex		*p*-value
		Female n_f_ = 121	Male n_f_ = 90	
**Comorbidity (4+)**	141 (66.8%)	93 (76.9%)	48 (53.3%)	0.0003
**Diabetes**	81 (38.4%)	45 (37.2%)	36 (40%)	> 0.1
**High blood pressure**	158 (73.0%)	90 (74.4%)	68 (66.7)	> 0.1
**Obesity**	15 (7.1%)	8 (6.6%)	7 (7.7%)	> 0.1
**Osteoporosis**	56 (26.3%)	50 (41.3%)	6 (6.7%)	< 0.0001
**Heart disease**	51 (24.2%)	30 (24.8%)	21 (23.3%)	0.07
**Gastrointestinal disorder**	43 (20.4%)	30 (24.8%)	13 (14.4%)	> 0.065
**Arthritis**	120 (56.9%)	84 (69.4%)	36 (40.0%)	< 0.0001
**Respiratory disease**	44 (20.9%)	29 (24.0%)	15 (16.7%)	> 0.1
**Visual**	166 (78.7%)	98 (81.0%)	68 (75.6%)	> 0.1
**Hearing**	36 (17.1%)	15 (12.4%)	21 (23.3%)	0.04
**Back pain**	118 (55.9%)	74 (61.2%)	44 (48.9%)	0.08
**Depression**	34 (16.1%)	22 (16.2%)	12 (13.3%)	> 0.01
**Anxiety**	37 (17.5%)	28 (23.1%)	9 (10.0%)	0.013
**Other**	79 (34.4%)	49 (40.5%)	30 (33.3%)	> 0.01^#^

^a^ Ratio between the number of reported conditions over the number of participants

^#^ Using Fisher exact test to compare sex distribution

### Prevalence of functional disabilities

Table 4 presents the estimated number of persons with physical function disability and type of physical function disability. The overall weighted prevalence of physical function disability using T-score among the study group was 58% (95% CI: 36, 49%). Therefore, we estimate that approximately 1560 subjects have some kind of physical function disability in the CMP. The estimated prevalence among females was 69.1% (95% CI: 60, 77%) and among males was 42.2% (95% CI: 32, 53%). Thus, the estimated number of persons with disability will be different by sex; approximately 1000 among females and 560 among males. The estimated prevalence showed different patterns by sex according to the index of functional disability. Based on the IADL, the estimated prevalence with physical function disability among females was 64.5% (95% CI: 56, 73%), and among males was 35.9% (95% CI: 26, 46%). Using Self-Care index, the estimated prevalence among females was 53.6% (95% CI: 45, 62%), and among males was 35.8% (95% CI: 26, 46%). And based on the Functional Mobility index the estimated prevalence among females was 60.4% (95% CI: 51, 69%) and among males was 35.9% (95% CI: 27, 47%).

**Table 4:** Estimated prevalence of persons with disability by type of physical function disabilities (*n* = 211)

Physical function disabilities	Estimated Prevalence^a^	95% CI^a^	Estimated number of persons with disability^a^
**T-score**			
Female disability	69.1%	(60,77%)	1000
Male disability	42.2%	(32,53%)	560
Overall disability	58%	(51,64%)	1560
**IADL**			
Female disability	64.5%	(56,73%)	935
Male disability	35.5%	(26,46%)	465
Overall disability	52%	(45,59%)	1400
**Self-Care**			
Female disability	53.6%	(45,62%)	780
Male disability	35.8%	(26,46%)	460
Overall disability	46%	(39,53%)	1240
**Functional Mobility**			
Female disability	60.4%	(51,69%)	875
Male disability	35.9%	(27,47%)	475
Overall disability	50%	(43,57%)	1350

^a^Weighted estimates according to the sampling design

Table 5 presents the prevalence ratio (PR) to estimate the magnitude of association between sex and physical function disability, controlling for age, income, marital status, number of chronic conditions and sampling design. This analysis was performed using four types of unconditional logistic models. The first (unadjusted) and second (adjusted) models considered the whole study group, while model 3 included those persons with high impact on functional disabilities conditions, and model 4 included those persons with low-moderate impact on functional disabilities. Our purpose was to assess if the presence of musculoskeletal conditions is a modifier effect of the relationship between sex and physical function disability, even though the likelihood ratio test to assess interaction term was statistically significant (*p* > 0.05), but our sample size was small to assess interaction terms when we stratified by participants with at least one musculoskeletal conditions of high impact (arthritis, low back pain and osteoporosis). Based on model 2, the estimated prevalence of physical function disability using T-score among females was 2.70 (95% CI: 1.1, 5.1) times the estimated prevalence of physical function disability using T-score among males, when adjusting for age, income, marital status, number of chronic conditions and sampling design. When we estimated the ${PR}_{adjusted}^{Females\  vs\  Males}$ among those participants with musculoskeletal conditions of high impact in functional disability (model 3), this adjusted excess in the prevalence was 2.11(95% CI: 1.1,3.5) using the T-score. However, we estimated the ${PR}_{adjusted}^{Females\  vs\  Males}$ among those participants categorized with musculoskeletal conditions of low impact (model 4), and a no-significant excess (*p* > 0.1) in prevalence of functional disability was showed (${PR}_{adjusted}^{Females\  vs\  Males}$: 1.02, 95% CI: 0.2,6.7) using the T-score. When we compared the ${PR}_{adjusted}^{Females\  vs\  Males}$ by type of participants (low and high impact of musculoskeletal conditions), the ${PR}_{unadjusted}^{Females\  vs\  Males}$ (only controlling the effect of the sampling design), indicates that magnitude of the association (PR) is underestimated (1.64 overall vs 2.11 in high impact). These patterns were similar when other indexes were used.

**Table 5:** Prevalence ratio (PR) of functional disability between sexes by different indexes

		Logistic Regression Model			
		Univariate Model with all subjects	Multivariate Models with all subjects	Multivariate Models in participants with high impact	Multivariate Models participants with low-moderate impact
Functional disability index	Sex	${PR}_{unadjusted}^{Fem\ vs\ Males}$ ^(1)^ (95% CI) (95% CI)	${PR}_{adjusted}^{Fem\ vs\ Males}$^(2)^ (95% CI)	${PR}_{adjusted}^{Fem\ vs\ Males}$ ^(3)^ (95% CI)	${PR}_{adjusted}^{Fem\ vs\ Males}$ ^(4)^ (95% CI)
T-score	Male	1	1	1	1
	Female	1.64 (1.2,2.1) ^a^	2.70 (1.4,5.1) ^a^	2.11 (1.1,3.5) ^b^	1.02 (0.2,6.7)
**IADL**	Male	1	1	1	1
	Female	1.82 (1.3,2.5) ^a^	3.64 (1.1,4.4) ^a^	2.63 (1.2,5.7) ^b^	0.95 (0.4,2.3)
**Self-Care**	Male	1	1	1	1
	Female	1.50 (1.1,2.1) ^b^	3.29 (1.1,9.9) ^b^	3.42 (1.3,5.8) ^a^	1.85 (0.1,7.4)
**Functional Mobility**	Male	1	1	1	1
	Female	1.68 (1.2,2.3) ^a^	2.81 (1.3,5.7) ^a^	2.60 (1.1,4.5) ^b^	1.04 (0.2,6.9)

^a^ (*p* < 0.001), ^b^ (*p* < 0.05), ^(*)^ No significant interaction terms were found in the model (*p* > 0.05),

^(1)^ Unadjusted for any potential confounders (only the sampling design was taking into account)

^(2)^ Adjusted for age, income, marital status, number of chronic conditions and sampling design

^(3)^ Adjusted for age, income, marital status and sampling design; among participants with high impact on functional disabilities

^(4)^ Adjusted for age, income, marital status and sampling design; among participants with low-moderate impact on functional disabilities

## Discussion

This study aimed to determine the prevalence of functional disabilities among Hispanics 65 years and older and to compare the prevalence of functional disabilities in specific self-care activities, IADLs and functional mobility between males and females; controlling for age, income, chronic conditions, and marital status. Two important findings were revealed in this study’s in-depth analysis. First, 58% of the Hispanic sample of older adults living in eight low-income communities in PR, had some type of physical function disability, with a higher prevalence among older females. Second, older female had each of the three disability types in a significant higher crude prevalence ratio (*p* < 0.05) than older males, that increased when adjusted for the sociodemographic variables.

The overall prevalence of physical function disability reported in the sample of this study (58%) is higher when compared to national and local data of representative samples with Hispanic populations. For example, data from the 2018 American Community Survey reported a disability prevalence 17.6% of independent living disability among Hispanic adults ≥65 years living in the USA and 26.6% among those Hispanic ≥65 years living in Puerto Rico [6]. The high burden of functional disabilities in our sample can be explained by their sociodemographic characteristics that have explained increased risk in disability in other studies such as low economic status [39–41], low educational levels [41–43], and high prevalence of having multiple chronic conditions [43, 44]. Direct comparison of our findings with previous studies should be undertaken with caution, as different measurement criteria, data collection methods, study populations and geographical parameters can greatly affect outcomes.

Female sex was associated with higher functional disability prevalence, a trend which has been widely reported across epidemiological studies. For example, global disability trends indicated by GBD 2017 [10] show that female individuals have had and continue to experience higher levels of disability than male individuals. At the national level, similar trends exist, with older females having a higher prevalence of independent living functional disabilities (16.4%) compared to males (10.7%) [6]. In Puerto Rico, disparities in independent living functional disabilities attributed to sex difference is more evident, with an estimated prevalence of 30.7% among females ≥65 years compared to 21.4% among male [6]. This disability disparity was even higher in our study group; the prevalence of functional disability among females was 2.70 (IC 95%: 1.4, 5.1) times the prevalence of functional disability among males when adjusting for age, income, chronic conditions, marital status, and sampling design. A consistent female disadvantage in self-care, IADL, and functional mobility domains remained significant even after adjusting for these co-variates, as seen in previous studies [9, 45, 46].

Moreover, the results of this study make a novel contribution concerning the modifier effect of having a musculoskeletal condition (arthritis, low back pain, and osteoporosis), in the assessment of functional disability and sex. This is, the effect of musculoskeletal conditions significantly changes (*p* < 0.05) the statistical association between the level of functional disabilities and sex. This association was not significant (*p* > 0.05) among the participants with other types of conditions such as high blood pressure, obesity, heart disease, gastrointestinal disorder, respiratory disease, visual, hearing, depression, or anxiety. These results point to the need of taking preventive actions to ensure the functional health of the specific sub-group of older women with musculoskeletal disorders.

Sex differences in functional disabilities among older adults with musculoskeletal conditions could be explained by personal as well as by cultural-related factors concerning gender roles. First, older females from the studied community had a significant higher prevalence of having four or more conditions compared to males, suggesting a poorer functional health status than their male counterparts. Second, there is some evidence supporting that arthritis, osteoporosis, and chronic back pain are more disabling for females than for males [9, 33–36]. It has also been reported a higher incidence, prolonged duration of functional disabilities, and faster decline in function over time in females compared with males [46, 47]. Third, the self-report nature of the data of physical function difficulties in our study raises the question of whether females may have similarly over reported (or males underreported) the levels of difficulties in daily living tasks, as seen in previous studies [48, 49], which may have resulted in false findings regarding sex differences. It has been suggested previously that females may find it more socially acceptable to report disability, whereas males are socialized to ignore them [50, 51]. On the other hand, in a study of sex differences that compared self-reported disability with performance measures concluded that males and females generally report their functional disabilities accurately, and the higher prevalence of functional problems among females may be a reflection of their true disability status [50].

Sex inequalities in IADL disability need to be interpreted in light of the gender specific roles and nature of IADLs activities in Hispanic culture. This is, Hispanic male is traditionally the economic provider while the Hispanic female is responsible for the household chores and caretaking roles [52–54]. This is particularly true for the older population of Hispanics. This gender role expectation results in an unequal distribution of household labor, with a higher involvement of females in household chores and preparing meals compared to males [54] which could in turn plausibly affect females’ physical health resulting in functional disabilities. Moreover, the female role of *Marianismo* that tends to stress self-denial, encourage females to subordinate their own health and prioritize the care of their family members – meanwhile ignoring signals of pain and illness in their own bodies and delaying medical attention [52, 53].

Further findings from this study suggests that females had higher reported functional disabilities in self-care and functional mobility compared to males, as seen in previous studies [9, 55]. Given that self-care activities (i.e. bathing and dressing) and functional mobility (i.e. walking, climbing stairs, and getting off the toilet) are gender neutral activities, these results may suggest that females are truly at a greater functional disadvantage compared to their male counterparts. These results are consistent with findings among older adults in previous studies [9, 45, 55] and support the hypothesis that a poorer functional health status and a higher impact of musculoskeletal conditions on function among females than males increase the magnitude of the sex . This sex gap could plausibly be explained by the impact of gender roles in the performance of daily activities in which women traditionally over-perform gender-specific tasks (e.g. household chores) which in turn may exacerbate chronic conditions such as arthritis, back pain, and osteoarthritis. Further research should be conducted to detangle the effects of sex and gender on functional disabilities.

Considering the greater functional disabilities of females in our study and the compensatory potential of assistive devices to increase older people independence, safety, and quality of life [56–58], this study highlights a finding with important implication concerning the use of assistive devices by females. We should expect greater assistive technology devices (i.e. canes, dressing sticks, or elevated toilet seats) needs and use among women as compared to males, as seen in previous studies [59–61]. Therefore, the findings of this study are of importance for future planning and development of policy to improve assistive technology access, particularly among the most vulnerable population of older women with physical function disabilities living in low-income communities.

Another important finding of this study is the role of the co-variates in altering the magnitude of the association between sex and functional disability within different populations. In a study conducted with 412 females and 328 males residing in underprivileged communities in Lebanon, the magnitude of the association between sex and disability in activities of daily living (self-care), IADL, and physical tasks (functional mobility), was decreased or even non-significant for ADL after adjusting for age, chronic disease risks factors, number of co-morbidities, prescription of medications, level of education, and marital status [55]. In contrast, in our study with an underprivileged community in Puerto Rico, a significantly increase in the magnitude of the association between sex and functional disability resulted after adjusting for age, income, chronic conditions and sampling design. This could be explained by differences in how the adjusted variables were measured. For example, in our study, we adjusted for two groups of co-morbidities (0–3 and ≥ 4) and in the Lebanon study they adjusted for the number of co-morbidities. Moreover, in our study we adjusted for the sampling design, an effect that was not reported on the Lebanon study. Adjustment for age was also different in the two samples (60 years and older in the Lebanon study versus 65 years and older in our study). Therefore, direct comparison of our findings with previous studies should again be undertaken with caution, as different covariates, as well as ways of measuring these co-variates can greatly affect outcomes.

### Strengths and limitations

This study has some limitations. First, the use of self-reported data introduces uncertainty about subjective interpretation of the questions. This may be influenced by the interviewee’ understanding of the question, as well as their experiences, expectations, and culture resulting in self-report bias. Therefore, we were unable to confirm actual physical function disabilities. However, studies have shown that self-reported disability is highly correlated with observed performance on similar tasks with no significant sex differences in reporting accuracy [50, 62]. Second, using a cross-sectional epidemiological design precludes drawing conclusions about cause-and-effect relationships; in addition, we expected some unbalance distribution by different characteristics in our data, thus, we decided to include different confounding variables based on our findings and according to scientific literature of potential risk factors for disability. Third, due to the COVID-19 pandemic we were unable to recruit the planned sample of 250 participants sample size. However, with this sample size the statistical power of the study was not affected.

The main strength of this study is the use of a population-based study using a random sample of residents in a low-income community. This study examined three kinds of physical function disability- self-care, IADL, and functional mobility. Collecting data from the community allows us to adjust for sociodemographic factors to determine potential confounding variables and modifiers effects. However, a longitudinal study is certainly needed to address health and physical function disability transitions, and the causal relationships between variables.

## Conclusions

The results of this study suggests that in PR, as in other parts of the world, females from low-income communities bear the greater burden of physical function disability in their adult age. Even though the sample of this study was homogenous with regard to race/ethnicity, geographical location, education, and income, which could limit the generalizability of these study findings, it served as a built-in control that supported our capacity to draw inferences from the sex comparisons. Given that underlying musculoskeletal conditions account in part for the sex differences in physical function disabilities, and the potential of adopting self-management behaviors to improve people with chronic conditions function, health, and quality of life [62], health policies as well as future studies should be targeted at reducing the burden of these nonfatal disabling conditions on function through disability self-management rehabilitation services that take into consideration sex and gender differences in functional disabilities among different types of daily activities. These rehabilitation services should be targeted at improving patient disability self-management behaviors, such as using assistive technology devices, environmental modifications, energy conservation techniques, and proper body mechanics, to compensate for the physical impairments inherent to the normal process of aging or having a chronic condition. The findings of this study might also inform public health programs of the sociodemographic characteristics and disparities in functional disabilities associated with age and sex. This data will guide efforts to improve the specificity and effectiveness of interventions, accessibility, and outreach to rehabilitation services to reduce the disability and sex disparities among the growing population of older adults. These targeted public health programs have the potential to improve health behaviors, prevent secondary conditions, and delay the deterioration of functional disabilities. Moreover, this study lends support to the importance within the primary health care services context to routinely inquire about physical function disabilities, particularly in the most vulnerable group of older people to direct appropriate and sensitive preventive and management programs for community-living older adults.

## Data Availability

The datasets used and/or analyzed during the current study are available from the corresponding author on reasonable request.

## Abbreviations

CI: Confidence interval
CMP: Caño Martín Peña
CT: Census tracks
IADL: Instrumental activities of daily living
PR: Prevalence ratio
SD: Standard deviation
USA: United State of America
